# Coping strategies, optimism, and resilience factors associated with mental health outcomes among medical residents exposed to coronavirus disease 2019 in Qatar

**DOI:** 10.1002/brb3.2320

**Published:** 2021-08-03

**Authors:** Malek Smida, Mohamed Adil Shah Khoodoruth, Saleem Khaldoon Al‐Nuaimi, Zerak Al‐Salihy, Adeel Ghaffar, Widaad Nuzhah Chut‐kai Khoodoruth, Mohammed Faisal Hamad Mohammed, Sami Ouanes

**Affiliations:** ^1^ Department of Psychiatry Hamad Medical Corporation Doha Qatar; ^2^ Graduate Medical Education Hamad Medical Corporation Doha Qatar; ^3^ Centre of Disease Control and Prevention Department Hamad Medical Corporation Doha Qatar

**Keywords:** coping, COVID‐19, medical residents, optimism, resilience

## Abstract

**Objective:**

The aim of this study is to examine the association between coping strategies, resilience, optimism and different mental health outcomes like stress, anxiety, and depression among the medical residents’ during the COVID‐19 pandemic, with consideration of different factors like seniority, frontliner, gender, and coping style.

**Methods:**

An electronic survey was sent to all medical residents in Qatar. Depression, anxiety, and stress were assessed by the DASS‐21. Professional quality of life was measured by the ProQOL scale. The coping mechanisms were assessed with the Brief‐COPE, and resilience was measured by the Brief Resilience Scale.

**Results:**

The most commonly used coping strategies were acceptance, religion, and active coping. The avoidant coping style scores were higher among junior residents (*p* = .032) and non‐COVID‐19 frontliners (*p* = .039). Optimism LOT‐R score was higher in senior than in junior residents (*p* < .001). Lower avoidant coping scores, higher optimism, and higher resilience were associated with lower stress, anxiety, and depressive symptoms.

**Conclusion:**

It seems that avoidant coping styles can exacerbate depressive, anxiety, and stress symptoms in medical residents amidst the COVID‐19 pandemic. Strategies promoting optimism, resilience, and approach coping styles can decrease the mental health burden of the pandemic on medical residents.

## Abstract 2

Avoidant coping styles can exacerbate depressive, anxiety, and stress symptoms in medical residents amidst the COVID‐19 pandemic. Strategies promoting optimism, resilience, and approach coping styles can decrease the mental health burden of the pandemic on medical residents.

## 1 BACKGROUND

Medical Residents in training represent a significant proportion of the medical staff within teaching hospitals all over the world. They are responding rapidly to changes in healthcare delivery during the COVID‐19 pandemic and this has had an impact on their training such as disruption of the regular annual schedule, deployment of residents to high‐risk areas and shifting educational activities online (Potts, 2020). Like many other doctors and healthcare workers in this situation, they too had fears for their own health and well‐being when caring for large numbers of infectious patients with a poorly understood disease (Nasrallah et al., 2020). Moreover, transmitting the virus to their household adds to their worry as health care workers and their families account for one in six (17.2%) hospital admissions for COVID‐19 (Shah et al., 2020). Qatar had to centralize their pool of medical residents for deployment to hospitals handling these cases (Nasrallah et al., 2020). Many residents were thus taken out of their specialty training to care for those in the isolation, intensive care units (ICU), and pneumonia wards. Others stayed working in their speciality's rotations, which led them to be divided into frontlines and second liners residents.

Medical residents are among the most vulnerable health care workers even in usual times with high prevalence of burnout and psychological morbidity (Prins et al., 2007). It was also mentioned that medical trainees exhibit high levels of depression, anxiety and stress compared to general population (Moir et al., 2018). A study conducted in Qatar on the same sample of medical residents used in this current study, showed that the prevalence of depression, anxiety, and stress were 42.5%, 41.7%, and 30.7%, respectively (Khoodoruth et al., 2021).

Nevertheless, the data about the impact of COVID‐19 on residents’ mental health outcomes remain poorly investigated even though, the current dramatical change during the pandemic on the residency program and lifestyle is likely to adversely impact their mental and physical well‐being (Abdessater et al., 2020; Civantos et al., 2020; Scott et al., 2020 ). In this context, coping skills are of a crucial importance in moderating the potential effects of the pandemic on one's mental health. However, research about how coping strategies and well‐being factors are related with depression, anxiety and stress among medical residents has not been conducted to the best of our knowledge.

A recent large cross‐sectional Chinese study performed among health workers demonstrated that factors like being a female, frontliner, having an intermediate technical title, and exposure to high‐risk areas were associated with worse mental health outcome (Lai et al., 2020). It is unclear how factors such as seniority in residency and exposure to high‐risk areas affect the psychological and coping responses among medical residents during the current pandemic. One Singaporean study has shown that no differences were found between junior and senior residents in terms of psychological and coping responses to the pandemic; however, data are still poorly investigated regarding the same purpose (Chew et al., 2020). An understanding of COVID‐19‐related psychological outcomes could highlight areas where better psychological support can be provided for our medical residents.

The psychological distress is associated with medical errors and lapses in professionalism as well as high risk of serious psychiatric problems including suicidal ideation (Van der Heijden et al., 2008; West et al., 2006 ); therefore, it is important to shed the light on the coping strategies and the factors, which could impact them to ensure the mental well‐being of the medical residents. Previous studies have shown that psychological resilience may mitigate the adverse effect of stress, and reduce the risk of depression and anxiety (Poole et al., 2017; Schulz et al., 2014 ). Optimism was also noted as an important ingredient helping people to build resilience and self‐efficacy, and among optimists, there were fewer reports on stress, less use of avoidance strategies and more practical focus on problem solving and search for social support (Dougall et al., 2001). As resilience and optimism might be protective factors for the mental well‐being of the medical residents as well, the assessment of these parameters with appropriate tools could prevent behavioral response and psychological distress during the pandemic.

In addition, screening tests regarding the mental health well‐being and/or symptoms might help the residency programs to implement interventional programs.

To address this gap, the present study aimed to assess the coping strategies, resilience and optimism factors in the medical residents’ workers during the COVID‐19 pandemic. It also aimed to evaluate the relationship between each of the variables (seniority, front liner, gender, coping style) and the symptoms of stress, depression, and anxiety.

## 2 METHODS

### 2.1 Setting and participants

A cross‐sectional survey was carried out, involving all medical residents from all specialties in Qatar (all residency programs in Qatar are under Hamad Medical Corporation). The survey took place from May 17, 2020 to June 16, 2020, amidst the COVID‐19 outbreak in Qatar.

### 2.2 Data collection and instruments

An email was sent to the professional email addresses of all medical residents in Qatar with a link to the survey. The survey was built using Qualtrics software (Qualtrics, Provo, UT, USA). In order to enhance participation rates, reminder emails were sent after one week, two weeks, and three weeks from the initial email.

The survey consisted of the following:

- Questions about the general characteristics of the sample.
- The Depression, Anxiety, and Stress Scale (DASS‐21), which corresponds to the shorter (21 items) of the original 42‐item DASS. DASS‐21is a well‐established instrument to examine symptoms of depression, anxiety and stress. Scores for each of these axes are obtained, with higher scores indicating higher levels of symptoms (Antony et al., 1998).
- The Brief‐COPE, which is a 28‐item questionnaire which assesses coping mechanisms. Each item consists of Likert scale ranging from 1 (I haven't been doing this at all”) to 4 (“I've been doing this a lot”). Each two items measure one of the following coping strategies: self‐distraction, active coping, denial, substance use, use of emotional support, use of instrumental support, behavioral disengagement, venting, positive reframing, planning, humor, acceptance, religion, and self‐blame. A score for each two overarching coping styles (avoidant coping vs. approach coping) can be obtained by summing up the subscales of the corresponding coping strategies. Religion and humor subscales are considered separately as they are not included in the calculation of the avoidant or approach coping scores (Carver, 1997)
- The Life Orientation Test‐Revised (LOT‐R), which is well‐established scale to assess dispositional optimism, defined as the general tendency to expect positive outcomes. The scale comprises 10 items: three items for optimism, three for pessimism (reverse scored), and four filler items (Hinz et al., 2017).
- The Brief Resilience Scale (BRS), which is a widely used scale to assess resilience as the ability to bounce back, with higher scores indicating higher resilience (Smith et al., 2008)

### 2.3 Statistical analysis

All statistical analyses were done using IBM SPSS software version 26.

For descriptive statistics of categorical variables, it was determined absolute and relative frequencies. For descriptive statistics of continuous variables, the mean and the standard deviation were calculated. It was also determined the median and interquartile range for nonnormally distributed variables (as per the Shapiro–Wilk's test).

The *t* test was used for independent samples to compare the Brief‐COPE, LOT‐R, and BRS scores between groups.

To assess the associations between DASS‐21 (depression, anxiety, and stress scores), Brief‐COPE, LOT‐R, and BRS scores, the nonparametric (Spearman's) correlations were used. A three‐way multivariate analyses of covariance (MANCOVA) was then constructed, using DASS‐21 depression, anxiety, and stress scores as dependent variables, and “residency seniority,” “being a frontliner or a second‐liner,” gender, Brief‐COPE, LOT‐R, and BRS scores as independent variables. Preliminary assumptions for MANCOVA (including normality, linearity, univariate and multivariate outliers, covariance matrices, and multicollinearity) were tested. Pillai's trace test was used because the DASS‐21 scores violated the normality assumption. The effect size was assessed using the partial *η* squared.

Holm–Bonferroni's method was used to correct for multiple comparisons. The defined significance level *α* was .05.

## 3 RESULTS

The response rate was 20% (*n* = 127 of 640 residents). Around two third of the respondents (63%, *n* = 79) were male, with 56% (*n* = 71) junior residents, and 63% (*n* = 80) being COVID‐19 frontliners. Table 1 shows the scores of depression, anxiety, stress, coping, optimism, and resilience among medical residents. The most commonly used coping strategies were acceptance, religion, and active coping. The least reported coping strategies were substance use and denial.

**TABLE 1:** Depression, anxiety, stress, coping, optimism, and resilience scores among medical residents

	Mean ± *SD*	Median	IQR
DASS‐21 depression	4.9 ± 4.5	4.0	7.0
DASS‐21 anxiety	3.6 ± 3.3	3.0	5.0
DASS‐21 stress	6.2 ± 4.2	5.0	6.0
Coping—self‐distraction	5.1 ± 1.6	5.0	2.0
Coping—active coping	5.1 ± 1.6	5.0	2.0
Coping—denial	2.9 ± 1.5	2.0	2.0
Coping—substance use	2.4 ± 1.2	2.0	1.0
Coping—emotional support	4.5 ± 1.6	4.0	2.0
Coping—use of informational support	4.1 ± 1.6	4.0	2.0
Coping—behavioral disengagement	3.6 ± 1.7	3.0	2.0
Coping—venting	4.0 ± 1.5	4.0	2.0
Coping—positive reframing	4.8 ± 1.7	5.0	2.0
Coping—planning	5.0 ± 1.6	5.0	2.0
Coping—humor	4.7 ± 1.9	4.0	2.0
Coping—acceptance	5.8 ± 1.6	6.0	2.0
Coping—religion	5.4 ± 1.9	6.0	3.0
Coping—self‐blame	4.0 ± 1.8	4.0	3.0
Avoidant coping style	22.0 ± 6.1	22.0	9.0
Approach coping style	29.3 ± 6.9	30.0	10.0
LOT‐R score	13.6 ± 4.2	14.0	5.0
BRS score	3.2 ± 0.7	3.2	1.0

BRS: Brief Resilience Scale; DASS‐21: Depression, Anxiety, and Stress Scale; IQR: interquartile range; LOT‐R: Life Orientation Test‐Revised; *SD*: Standard deviation.

Avoidant coping style scores were higher among junior residents (22.99 ± 7.09 vs. 20.77 ± 4.34, *p* = .032) and non‐COVID‐19 frontliners (23.47 ± 5.79 vs. 21.15 ± 6.17, *p* = .039). Optimism LOT‐R score was higher in senior than in junior residents (12.25 ± 4.40 vs. 15.25 ± 3.28, *p* < .001) (Table 2).

**TABLE 2:** Comparison of depression, anxiety, stress, coping, optimism, and resilience scores a between junior versus senior medical residents and between COVID‐19 frontliners and nonfrontliners

	Junior residents	Senior residents	*p*	Non‐COVID‐19 frontliners	COVID‐19 frontliners	*p*
DASS‐21 depression	5.54 ± 5.17	4.11 ± 3.48	.066	5.32 ± 4.54	4.66 ± 4.55	.434
DASS‐21 anxiety	3.92 ± 3.46	3.18 ± 3.00	.209	3.85 ± 3.01	3.44 ± 3.43	.494
DASS‐21 stress	6.87 ± 4.56	5.45 ± 3.67	.053	6.81 ± 4.30	5.91 ± 4.19	.251
Coping—humor	4.93 ± 2.11	4.43 ± 1.61	.131	4.85 ± 1.76	4.62 ± 2.00	.522
Coping—religion	5.11 ± 1.95	5.68 ± 1.77	.094	5.57 ± 1.84	5.24 ± 1.92	.334
Avoidant coping style	**22.99 ± 7.09**	**20.77 ± 4.34**	**.032**	**23.47 ± 5.79**	**21.15 ± 6.17**	**.039**
Approach coping style	28.65 ± 7.53	30.16 ± 6.03	.211	29.81 ± 7.44	29.03 ± 6.64	.540
LOT‐R score	**12.25 ± 4.40**	**15.25 ± 3.28**	**.000**	13.72 ± 4.35	13.49 ± 4.14	.762
BRS score	3.17 ± 0.74	3.32 ± 0.68	.233	3.13 ± 0.74	3.30 ± 0.69	.189

All values are represented as mean ± standard deviation.

BRS: Brief Resilience Scale; DASS‐21: Depression, Anxiety, and Stress Scale; LOT‐R: Life Orientation Test‐Revised; Bold indicates statistical significance.

**TABLE 3:** Nonparametric correlations between depression, anxiety, stress, coping, optimism, and resilience scores among medical residents

		DASS‐21 depression	DASS‐21 anxiety	DASS‐21 stress	Coping—humor	Coping—religion	Avoidant coping style	Approach coping style	LOT‐R score
DASS‐21 anxiety	*ρ*	**.592**							
	*p*	**.000**							
DASS‐21 stress	*ρ*	**.693**	**.706**						
	*p*	**.000**	**.000**						
Coping—humor	*ρ*	.159	**.261**	.196					
	*p*	.411	**.013**	.186					
Coping—religion	*ρ*	−.031	.059	.055	.159				
	*p*	1.000	1.000	1.000	.443				
Avoidant coping style	*ρ*	**.533**	**.546**	**.553**	**.292**	.104			
	*p*	**.000**	**.000**	**.000**	**.013**	.443			
Approach coping style	*ρ*	−.158	.096	.013	**.213**	**.460**	.209		
	*p*	.443	.443	1.000	**.040**	**.000**	.176		
LOT‐R score	*ρ*	−**.498**	−**.323**	−**.367**	−.168	.196	−**.345**	**.334**	
	*p*	**.000**	**.000**	**.000**	.248	.248	**.000**	**.000**	
BRS score	*ρ*	−**.314**	−**.258**	−**.309**	−.135	−.030	−**.378**	.114	**.413**
	*p*	**.000**	**.039**	**.000**	.443	1.000	**.000**	.443	**.000**

*Note: p* Values are adjusted for multiple comparison using Holm–Bonferroni's method.

BRS: Brief Resilience Scale; DASS‐21: Depression, Anxiety, and Stress Scale; LOT‐R: Life Orientation Test‐Revised. Bold indicates statistical significance.

### 3.1 Correlations between depression, anxiety, stress, coping, optimism, and resilience scores among medical residents (Table 3)

There were moderate positive bivariate correlations between all three DASS‐21 scores (*ρ* values ranging between .592 and .706, *p* values < .001).

DASS‐21 depression score was positively correlated with avoidant coping style (*ρ* = .533, *p* < .001), and negatively correlated with optimism and resilience scores (*ρ* = –.498, *p* < .001; and *ρ* = –.314, *p* < .001, respectively).

DASS‐21 anxiety score was positively correlated with humor and avoidant coping style (*ρ* = .261, *p* = .013 and *ρ* = .546, *p* < .001, respectively) and negatively correlated with optimism and resilience scores (*ρ* = –.323, *p* < .001 and *ρ* = ‐.258, *p* < .001, respectively).

DASS‐21 stress score was positively correlated with avoidant coping style (*ρ* = .553, *p* < .001) and negatively correlated with optimism and resilience scores (*ρ* = –.367, *p* < .001 and *ρ* = –.309, *p* < .001, respectively).

### 3.2 Multivariate analysis (Tables 4 and 5)

The MANCOVA analysis showed significant effects of the avoidant coping style and of the LOT‐R optimism score on mental health outcomes (as measured by DASS‐21 depression, anxiety, and stress scores as dependent variables). The effect size was large (partial *η* ^2 ^= .185) for the avoidant coping style and medium (partial *η* ^2 ^= .112) for the LOT‐R score (Table 4).

**TABLE 4:** Multivariate covariance analysis assessing the associations between mental health outcomes (DASS‐21) in medical residents, and their coping styles, optimism, and resilience

Effect	Pillai's trace	*F*	*p*	Partial *η* squared
Coping—humor	0.028	1.112	.347	.028
Coping—religion	0.003	0.097	.962	.003
Avoidant coping style	**0.185**	**8.698**	**.000**	**.185**
Approach coping style	0.038	1.517	.214	.038
LOT‐R	**0.112**	**4.850**	**.003**	**.112**
BRS	0.048	1.922	.130	.048
Gender	0.006	0.222	.881	.006
COVID‐19 frontliner	0.003	0.110	.954	.003
Seniority in residency	0.019	0.735	.533	.019

BRS: Brief Resilience Scale; DASS‐21: Depression, Anxiety, and Stress Scale; LOT‐R: Life Orientation Test‐Revised. Bold indicates statistical significance.

Univariate tests of between‐subjects effects (Table 5) showed that the avoidant coping score had a large effect size on the depression score, and medium effect sizes on anxiety and stress scores, with higher avoidant coping scores being associated with a worse outcome. The LOT‐R optimism score had a medium effect size on the depression score, but no significant effects on anxiety or stress scores.

**TABLE 5:** Univariate tests of between‐subjects effects with DASS‐21 scores as dependent variables

	Dependent variable	Type III sum of squares	Mean square	*F*	*p*	Partial *η* squared
Coping—humor	DASS‐21 depression	0.088	0.088	0.007	.933	.000
	DASS‐21 anxiety	20.395	20.395	2.404	.124	.020
	DASS‐21 stress	0.676	0.676	0.053	.819	.000
Coping—religion	DASS‐21 depression	0.819	0.819	0.067	.796	.001
	DASS‐21 anxiety	0.271	0.271	0.032	.858	.000
	DASS‐21 stress	1.085	1.085	0.084	.772	.001
Avoidant coping style	**DASS‐21 depression**	**286.722**	**286.722**	**23.482**	**.000**	**.167**
	**DASS‐21 anxiety**	**119.325**	**119.325**	**14.062**	**.000**	**.107**
	**DASS‐21 stress**	**180.270**	**180.270**	**14.038**	**.000**	**.107**
Approach coping style	DASS‐21 depression	35.327	35.327	2.893	.092	.024
	DASS‐21 anxiety	0.013	0.013	0.002	.968	.000
	DASS‐21 stress	0.148	0.148	0.012	.915	.000
LOT‐R	**DASS‐21 depression**	**172.042**	**172.042**	**14.090**	**.000**	**.107**
	DASS‐21 anxiety	13.769	13.769	1.623	.205	.014
	DASS‐21 stress	38.632	38.632	3.008	.085	.025
BRS	DASS‐21 depression	15.264	15.264	1.250	.266	.011
	DASS‐21 anxiety	1.265	1.265	0.149	.700	.001
	**DASS‐21 stress**	**59.556**	**59.556**	**4.638**	**.033**	**.038**
Gender	DASS‐21 depression	0.001	0.001	0.000	.994	.000
	DASS‐21 anxiety	0.015	0.015	0.002	.967	.000
	DASS‐21 stress	4.360	4.360	0.340	.561	.003
COVID‐19 frontliner	DASS‐21 depression	0.179	0.179	0.015	.904	.000
	DASS‐21 anxiety	0.649	0.649	0.077	.783	.001
	DASS‐21 stress	0.479	0.479	0.037	.847	.000
Seniority in residency	DASS‐21 depression	10.725	10.725	0.878	.351	.007
	DASS‐21 anxiety	0.716	0.716	0.084	.772	.001
	DASS‐21 stress	0.878	0.878	0.068	.794	.001

BRS: Brief Resilience Scale; DASS‐21: Depression, Anxiety, and Stress Scale; LOT‐R: Life Orientation Test‐Revised. Bold indicates statistical significance.

## 4 DISCUSSION

Even though the response rate for the study survey was relatively low (20%) compared to the percentage rate of participants in another similar study in Singapore (response rate of 49.2%) (Chew et al., 2020), it tried to analyze different variables that could correlate with the coping mechanisms.

The most used coping strategies in our study were acceptance, religion, and active coping. The least reported ones were substance use and denial. Avoidant coping style scores were higher among junior residents in our study. However, optimism score was higher in senior than in junior medical residents. In this study depression, anxiety, and stress were positively correlated with avoidant coping style. The avoidant coping has large effect size on the depression score and medium effect size on anxiety and stress, with higher avoidant coping score being associated with a worse outcome. There were no differences for coping style effect on mental health outcome regarding gender, seniority, and frontliner in our study. Optimism was found to be significantly correlated with depression with a medium effect size, but there was no significant correlation with anxiety or stress. Finally, resilience of the medical residents was negatively associated with depression, anxiety, and stress.

### 4.1 Coping strategies

Coping behaviors and strategies have been traditionally dichotomized into categories, such as problem‐ versus emotion‐focused, functional versus dysfunctional, approach versus avoidance, engagement versus disengagement, and primary versus secondary control coping (García et al., 2018). Carver categorizes the strategies of acceptance, emotional social support, humor, positive reframing, and religion as emotion focused (Carver, 1997). On the other hand, active coping, instrumental support, and planning are considered as problem‐focused strategies. Finally, behavioral disengagement, denial, self‐distraction, self‐blaming, and substance use and venting are considered as dysfunctional coping strategies. Considering that coping strategies may be classified as adaptive or maladaptive depending on different factors, there are sufficient empirical evidence that point out which are the most commonly related to emotional distress or well‐being. The most used coping strategies in our study were acceptance, religion, and active coping. The least reported ones were substance use and denial. These findings are similar to one study conducted to medical residents in Saudi Arabia in 2015 (Alosaimi et al., 2015). A Chinese study in Hong Kong during the outbreak of severe acute respiratory syndrome (SARS) among emergency medical staff (nurses and doctors) showed that the most common coping strategies used are acceptance, active coping, and positive framing while working. However, religion was less likely to be used (Wong et al., 2005).

The most commonly used coping mechanisms by medical residents in Qatar are the adaptive ones. Indeed, coping strategies measured by Brief‐COPE have been classified into maladaptive coping, which including venting, denial, substance use, behavioral disengagement, self‐distraction, and self‐blame, and adaptive coping, including positive reframing, planning and seeking social support, active coping, use of emotional and instrumental support, acceptance, religion, and humor (Meyer, 2001).

Unlike the limited role of religion as a coping strategy among residents and physicians in the United States (Sargent et al., 2004; Taft et al., 2011 ), religion was one of the most frequently used adaptive stress‐coping strategies in the current study. This may reflect the critical role of religion in all aspects of behavior in a conservative country such as Qatar.

However, religion has been considered in some studies as a maladaptive strategy, whereas in other studies appears with an adaptive value (García et al., 2018). The possible reported reason being variability in religiosity and spirituality among individuals.

There were no differences found between junior and senior residents in terms of coping responses in the Singaporean study (Chew et al., 2020). However, in our study avoidant coping style scores were higher among junior residents. This result may be due to a lack of work experience in similar stressful situations. Another possible reason is that, during the current pandemic, the lack of health care staff has required that senior residents or people with fewer experience have had to deal with the demands of the COVID‐19 patients.

The avoidant coping style was also lower in frontliners. This may be explained by the higher level of psychological preparedness in those deployed to high‐risk areas. Those deployed to COVID sites are aware that they will be screening and managing patients with suspected or confirmed COVID‐19 infection in a well‐equipped facility with ample staff support and training. Recent research indicates that one of the greatest concerns of health personnel is the possibility of infecting others, especially family members (Adams & Walls, 2020). Believing that they are very unlikely to be infected with COVID‐19 is related to fewer symptoms of posttraumatic stress and anxiety; therefore, the avoidant behavior would be less for the frontlines.

One previous study has shown that gender differences exist regarding the ability to cope with stress (Eisenbarth, 2019). A higher tendency of females in different populations to use maladaptive or emotion‐focused stress‐coping strategies and to have more stress have been previously reported (Al‐Sowygh, 2013; Levey, 2001; Taft et al., 2011). However, in our study, there was no gender differences regarding coping styles, which could be explained by all females and males medical residents during COVID‐19 pandemic have the same stress levels.

### 4.2 Optimism

One of our fields of interest in this study is to shed the light on optimism factors among the medical residents during the COVID‐19 pandemic, as optimism is considered one of the coping mechanisms (World Health Organization, 2009).

A clear set of coping skills, including how to think optimistically and how to approach problems and adversities, has been shown to help the health care workers. Gaining the skill of optimism can assist in confronting stress or setback, can help to overcome failure in particular events, and strengthens self‐efficacy and resilience. This will increase the health care workers’ overall sense of well‐being, helping them to be more beneficial to their society (Seligman, 2004).

In this study, it has been found that optimism score was higher in senior than in junior medical residents. This finding may be explained by the difference of number of year experience between them.

A Swedish study among pediatric oncologists in 2009 showed that links were found between their level of optimism and experience, and their optimistic attitude was helpful for their resilience (Stenmarker et al., 2009). An Indian study among nurses performed during COVID‐19 pandemic indicated that psychological preparedness, self‐efficacy, resilience, and optimism were higher among the nursing faculty and administrators than the students, and it was explained by that the lack in these areas for the younger student might improve with age and work experience (Gandhi et al., 2020).

### 4.3 Mental health outcomes and coping factors

In our study, depression, anxiety, and stress positively correlated with avoidant coping style. Only anxiety was positively correlated with humor coping style. There is no difference for coping style effect on mental health outcome regarding gender, seniority, and frontliner in our study. The avoidant coping had large effect size on the depression score and medium effect size on anxiety and stress, with higher avoidant coping score being associated with a worse outcome.

The current findings may indicate that medical residents during COVID 19 pandemic while using inappropriate adaptive stress‐coping strategies such as avoidant can lead to higher chance to get mental health disease, whereas only some adaptive coping like humor is weakly associated with anxiety.

Similar to the current findings, maladaptive stress coping strategies in physicians have been linked to the presence of psychiatric disorders such as depression and anxiety (Hoonpongsimanont et al., 2014), which can be regarded as failure to adequately resolve stress caused by work (Bittner et al., 2011). Meyer found that maladaptive strategies have a greater relationship with mental health problems such as depression (Meyer, 2001). On the other hand, adaptive strategies have a stronger relationship with psychological well‐being. Accordingly, maladaptive strategies have been found to be related to perceived stress and adaptive ones to satisfaction with life (García et al., 2018).

Studies have shown that optimistic individuals have better physical and mental health than others (Carver & Scheier, 2017), which is consistent with our study in which we found that depression, anxiety, and stress were negatively correlated with optimism and resilience. Optimism was found to be significantly correlated with depression with a medium effect size, but there was no significant correlation with anxiety or stress.

Optimism has consistently been reported to be an indicator of more stress resilience, more coping mechanisms, and reduced depression (Ran et al., 2020). However, the same study indicated that optimism was correlated with less anxiety, which is not consistent with our study. In general, researchers have demonstrated that optimism has the power to improve morbidity outcomes and enhance team and organization performance during crisis (Carver & Scheier, 2017).

Consistent with our study, resilience of nonlocal medical workers in a Chinese study was negatively associated with depression and anxiety (Lin et al., 2020). The same study that examined resilience, anxiety, depression, and coping strategies among nonlocal medical workers sent to Wuhan to support local staff in treating patients infected with COVID‐19 found that resilience was associated with active coping, depression, anxiety, as well as training and support from the hospital (Lin et al., 2020). Cross‐sectional and longitudinal studies have confirmed that resilience reduced the risk of depression in individuals with adverse childhood experiences (Poole et al., 2017; Schulz et al., 2014 ). Furthermore, low resilience to stress during adolescence was associated with an increased risk of lifelong use of antidepressant and anxiolytic drugs (Hiyoshi et al., 2015). Thus, resilience is an essential buffer for stress or a traumatic incident and could protect against psychological distress. As such, the assessment of individual psychological resilience could help to predict mental health status.

### 4.4 Strengths and limitations

To the best of our knowledge, this study has been the first to examine coping strategies, resilience, and optimism factors and their associations with the mental health outcomes among medical residents during the COVID‐19 pandemic. Our study involved all medical residents in Qatar across all specialties. We also used well‐validated tools for assessment. Nevertheless, we acknowledge a number of limitations. First, the cross‐sectional design does not allow to examine changes over time or to draw any conclusions regarding any causal links. Second, we were not able to assess whether nationality (being expatriate or local resident) or specialty impacted coping and resilience factors due to the anonymous nature of the survey. Third, being a self‐reported study, the possibility of reporting bias, especially for culturally unacceptable coping strategies, cannot be excluded. Finally, the response rate of 20% may affect the generalizability.

### 4.5 Recommendations and conclusion

In conclusion, supporting the mental health of medical residents is not morally justified but if done well, it should augment their opportunity to experience psychological growth from overcoming the challenges faced during this pandemic. Residents should be appropriately thanked, for the acknowledgement of the hard work shouldered foster resilience (McCanlies et al., 2018). This appreciation should also include recognition of potential psychological difficulties, especially among less optimistic junior residents who displayed avoidant coping and provide information about support options available.

Therefore, the implementation by residency programs of several interventions that enhance the coping mechanisms is required. For instance, psychological preparedness, mindfulness, communication, and social support have been shown efficient strategies to reinforce resilience and optimism (Dougall et al., 2001; Gandhi et al., 2020; Rosenzweig et al., 2003; World Health Organization, 2009).

## FUNDING

Open access funding was provided by the Qatar National Library.

## ETHICAL APPROVAL

The study was approved by the Hamad Medical Corporation Institutional Review Board (MRC‐05‐049), and all study participants provided electronic consent.

## Data Availability

The data that support the findings of this study are available from the corresponding author (M.S.) upon reasonable request.
